# Extended Joinpoint Regression Methodology for Complex Survey Data

**DOI:** 10.1002/sim.70374

**Published:** 2026-01-22

**Authors:** Benmei Liu, Hyune‐Ju Kim, Joe Zou, Eric J. Feuer, Barry I. Graubard

**Affiliations:** ^1^ Division of Cancer Control and Population Sciences, National Cancer Institute Bethesda Maryland USA; ^2^ Department of Mathematics Syracuse University Syracuse New York USA; ^3^ Information Management Services, Inc. Calverton Maryland USA; ^4^ Division of Cancer Epidemiology and Genetics National Cancer Institute Bethesda Maryland USA

**Keywords:** complex survey data, individual‐level model, joinpoint regression, modified design‐based AIC, trend analysis

## Abstract

Joinpoint regression can model trends in time‐specific aggregated estimates. These methods have been developed mainly for non‐survey data such as cancer registry data, and only recently have been extended to utilize survey data that accounts for complex sample designs resulting in non‐zero correlation between the time‐specific estimates. This correlation can occur for surveys with data from the same sampled units used across time points, for example, the annual National Health Interview Survey with multistage cluster samples using the same first‐stage sampled clusters over consecutive time points. Another issue when modeling aggregated data is that the degrees of freedom for joinpoint analyses of multistage cluster samples are based on the number of time points, not the number of first‐stage sampled clusters as used in survey methods. To address this, we propose models of individual‐level data that incorporate both the correlation between time points and correct the degrees of freedom due to the sampling design that is needed for accurate inferences. Also, a modified design‐based Akaike Information Criterion (M‐dAIC) for model selection is proposed to account for complex sample designs. These new methods are empirically compared to existing methods using simulation studies and health survey data examples. The simulation studies indicated that this new individual‐level model identified the true number of joinpoints more accurately than the established aggregate‐level models for data collected using complex survey designs with moderate to large interclass correlation coefficients (ICC).

## 1 Introduction

Time trend analysis with data from complex surveys has been used by researchers and government agencies to identify patterns of change over time, to make predictions and to inform decision‐making on a variety of important socio‐economic and behavioral issues including health outcomes, for example, see [1, 2, 3, 4, 5]. Some of these time trend analyses have applied joinpoint regression models to aggregated outcomes, that is, observations grouped by time. For example, trend analysis of prevalence of ever smoking a cigarette among students ingrades 9–12 aggregated at each survey year [4], implemented in the National Cancer Institute's (NCI's) Joinpoint software [6].

Joinpoint regression is used to model linear trends in outcomes over continuous time, identifying abrupt changes in slope (i.e., joinpoints) where both the number and locations of these changes are estimated from the data. While we do not necessarily believe that trends change at a constant rate for a fixed period and then suddenly shift direction, this approach serves as an approximation of reality (as all models do) and has proven useful in interpreting these trends. In many cases, the changes in trends have been interpretable in terms of, for example, the introduction of new policies or innovations. The NCI Joinpoint software, used for conducting joinpoint regression with specific types of data, has been downloaded over 12 000 times a year. It has become the standard tool for interpreting population cancer trends and a wide range of other health‐related outcomes.

When applying the NCI Joinpoint software (prior to version 4.9) to repeated complex cross‐sectional surveys with stratified multistage cluster probability sample designs, there can be covariances between the surveys due to including the same first‐stage sampled units across time, that is, the same primary sample units (PSUs) are used in multiple years; an issue that does not arise from cancer registry data. This was addressed in the newer version (4.9+) of the NCI Joinpoint software with a feature to incorporate an estimated variance–covariance matrix for aggregated time‐specific data into joinpoint models [7]. However, when modeling aggregated data, as the input data is only a single value at each time point, the degrees of freedom for variance estimation for the joinpoint estimates are based on the number of time points, not the usually larger number of sampled PSUs that is used for degrees of freedom when analyzing individual‐level data from complex surveys. This is a point that has been raised because of potential loss in statistical power from the reduced degrees of freedom based on the number of time points [1, 8].

In this research, we propose joinpoint models of individual‐level data, which provide a more granular view of the data, incorporating both correlated errors and corrected degrees of freedom to account for the complex sampling design, ensuring accurate statistical inferences. We elaborate on the joinpoint methodology for individual‐level models and introduce a modified design‐based Akaike Information Criterion (M‐dAIC) for model selection, specifically tailored to account for complex sample designs and the nonlinear nature with additional unknown parameters of joinpoint models.

The rest of the paper is organized as follows: Section 2 briefly reviews the existing methods for aggregate‐level models. In Section 3, we propose individual‐level joinpoint models for both continuous and binary health outcomes and develop a modified design‐based M‐dAIC for joinpoint model selection. The proposed new methods are compared to aggregate‐level methods using simulation studies and empirical data analysis, presented in Sections 4 and 5, respectively. A summary and discussion of this paper are provided in Section 6.

## 2 Aggregate‐Level Models: A Review

In stratified multistage cluster probability sample designs the target finite population is partitioned into first‐stage clusters, PSUs, that are often geographically based such as counties or cities. The PSUs are grouped into disjoint sampling strata that are formed to be approximately homogeneous. For example, the PSUs are grouped with respect to specific demographic characteristics of the PSU‐level populations such as racial/ethnic group distributions. At the first stage of sampling, two or more PSUs are randomly sampled within each sampling stratum. At the second and subsequent stages, stratification and cluster sampling can be used to ultimately randomly sample individuals within each sampled PSU. For purposes of variance estimation, it is usually assumed that the first‐stage sampling of the PSUs can be well approximated by sampling with replacement and that sampling within the sampled PSUs is done independently between PSUs.

Let $y_{\mathrm{ijl}}^{\left(t\right)}$ be the outcome (either continuous or binary) of interest and $w_{\mathrm{ijl}}^{\left(t\right)}$ be the survey sample weight for each randomly sampled individual $l\;\left(l=1,\ldots ,n_{\mathrm{ij}}^{\left(t\right)}\right)$ in sampling stratum i (i=1,…,I) and sampled PSU j $\;\left(j=1,\ldots ,J_{i}^{\left(t\right)}\right)$ for a given time point t (t = $t_{1},\ldots ,t_{T}$). The $w_{\mathrm{ijl}}^{\left(t\right)}$ are the inverse of the probability of selection of the sampled individual ijl at time t, where these weights can include adjustments for nonresponse and post‐stratification or raking to target population totals.

Liu et al. [7] considered the following *k*‐joinpoint model at the aggregate level for trend analysis:

(1) $$g\left(y^{\left(t\right)}\right)=\beta_{A0}+\beta_{A1}t+\delta_{A1}\left(t-\tau_{1}\right)I_{\tau_{1}}\left(t\right)+\cdots +\delta_{\mathrm{Ak}}\left(t-\tau_{k}\right)I_{\tau_{k}}\left(t\right)+\varepsilon_{\mathrm{At}},$$

where $\;y^{\left(t\right)}$ is a sample weighted estimate of the $y_{\mathrm{ijl}}^{\left(t\right)}$ (e.g., total, mean or proportion) at each time point t (t = $\;t_{1},\ldots ,t_{T}|t_{1}<\cdots <t_{T}$), $\left\{\tau_{1},\ldots ,\tau_{k}|\tau_{1}<\cdots <\tau_{k}\right\}$ are the unknown joinpoints and $I_{\tau_{k}}\left(t\right)=1$ if $t>\tau_{k}$ and $I_{\tau_{k}}\left(t\right)=0$ if $t\leq \tau_{k}$, $\beta_{A0}$ is the intercept, $\;\beta_{A1},\delta_{A1},\ldots ,\delta_{\mathrm{Ak}}$ are the coefficient parameters, the error terms $\left(\varepsilon_{A1},\ldots ,\varepsilon_{\mathrm{AT}}\right)∼N\left(0,{⅀}_{T\times T}\right)$, and $g\left(y^{\left(t\right)}\right)=y^{\left(t\right)}$ or $g\left(y^{\left(t\right)}\right)=\log\left(y^{\left(t\right)}\right)$, the natural logarithm transformation of the aggregate‐level y at year t. For a *k*‐joinpoint model, there are *k* + 1 time segments $\left\{\left(\tau_{0}\tau_{1}\right],\left(\tau_{1}\tau_{2}\right],\ldots ,\left(\tau_{k}\tau_{k+1}\right]\right\}$, where $\tau_{0}=t_{1}$ and $\tau_{k+1}=t_{T}$ and segment s=1,…,k+1 is denoted by $\left(\tau_{s-1},\tau_{s}\right]$. For this application, we restrict $\left\{\tau_{1},\ldots ,\tau_{k}\right\}\subset \left\{t_{1},\ldots ,t_{T}\right\}.$ The parameterization in model (1) is called standard parameterization in the NCI Joinpoint software.

When a natural log‐scale transformation is used, that is, $g\left(y^{\left(t\right)}\right)=\log\left(y^{\left(t\right)}\right)$, the slope of the sth segment corresponds to annual percent change (APC). The model for a given segment s is assumed to be the line segment: $\log\left(y^{\left(t\right)}\right)=\beta_{s,A0}+\beta_{s,A1}t+\varepsilon_{\mathrm{At}}$ for times t in $\left(\tau_{s-1},\tau_{s}\right]$, where $\beta_{s,A0}$ and $\beta_{s,A1}$ denote the intercept and slope for segment s, s=1,…,k+1, respectively. Following this general parameterization, for the first segment (i.e., s=1), $\beta_{1,A0}=\beta_{A0}$, and $\beta_{1,A1}=\beta_{A1}$; for the *s*th segment (s>1), $\beta_{s,A0}=\beta_{A0}-\left(\delta_{A1}\tau_{1}+\ldots +\delta_{A\left(s-1\right)}\tau_{s-1}\right)$, and $\beta_{s,A1}=\beta_{A1}+\delta_{A1}+\ldots +\delta_{A\left(s-1\right)}.$

The estimated APC for the untransformed outcome y from year t to year t+1 in segment s can be derived as: ${\mathrm{APC}}_{\mathrm{sA}}=100\left[{\hat{y}}^{\left(t+1\right)}-{\hat{y}}^{\left(t\right)}\right]/{\hat{y}}^{\left(t\right)}=100\left(e^{{\hat{\beta }}_{s,A1}}-1\right),$ where ${\hat{\beta }}_{s,A0}$ and ${\hat{\beta }}_{s,A1}$ are the estimators of $\beta_{s,A0}$ and $\beta_{s,A1}$, respectively. The average APC (AAPC) is a summary measure of the trend over a pre‐specified fixed interval and is computed as a weighted average of the APCs from the joinpoint model, with the weights (denoted as $\gamma_{s})$ equal to the length of the APC interval s,s≥1. That is, $\;{\text{AAPC}}_{A}=100\left[\exp\left(\frac{\sum \gamma_{s}{\hat{\beta }}_{s,A1}}{\sum \gamma_{s}}\right)-1\right]$. The 95% confidence intervals of ${\mathrm{APC}}_{\mathrm{sA}}$ and ${\text{AAPC}}_{A}$ are provided at the Supporting Information Material.

A permutation test and several Bayesian Information Criterion (BIC) methods are used to determine the number and location of the joinpoints [9]. Liu et al. [7] accounted for the full variance–covariance matrix of the error terms.

## 3 Individual‐Level Models

In this section, we propose joinpoint methods based on individual‐level models with k (≥0) joinpoints to account for the degrees of freedom and the correlations between the time‐specific estimates from the complex survey sample design simultaneously.

### 3.1 Model Specification

For a continuous outcome $y_{\mathrm{ijl}}^{\left(t\right)}$, we consider the following joinpoint model at the individual level:

(2) $$g\left(y_{\mathrm{ijl}}^{\left(t\right)}\right)=\beta_{0}+\beta_{1}t+\delta_{1}\left(t-\tau_{1}\right)I_{\tau_{1}}\left(t\right)+\ldots +\delta_{k}\left(t-\tau_{k}\right)I_{\tau_{k}}\left(t\right)+\varepsilon_{\mathrm{ijl}}^{\left(t\right)};$$

where ${\tau_{k}}^{\prime }s$ are the unknown joinpoints with $I_{\tau_{k}}\left(t\right)=1$ if $t>\tau_{k}$; and $\varepsilon_{\mathrm{ijl}}^{\left(t\right)}$ is the error term, $\left(\varepsilon_{\mathrm{ijl},}^{1}{\ldots ,\varepsilon }_{\mathrm{ijl}}^{T}\right)∼N\left(0,{⅀}_{T\times T}\right)$ at given i,j,l. The link function g(·) can be either an identity or a natural log function. When identity function is chosen, that is, $g\left(y_{\mathrm{ijl}}^{\left(t\right)}\right)=y_{\mathrm{ijl}}^{\left(t\right)},$ model (2) could be reduced to model (1), though model (2) allows for inclusion of individual level covariates. For this study, we focus on the log function $g\left(y_{\mathrm{ijl}}^{\left(t\right)}\right)=\log\left(y_{\mathrm{ijl}}^{\left(t\right)}\right).$

For a binary outcome $y_{\mathrm{ijl}}^{\left(t\right)},$ we consider the following logistic joinpoint model:

(3) $$\begin{array}{cc} \text{logit}\left(p\left(y_{\mathrm{ijl}}^{\left(t\right)}=1\right)\right) & =\beta_{0}+\beta_{1}t+\delta_{1}\left(t-\tau_{1}\right)I_{\tau_{1}}\left(t\right)+\ldots \\ & \;+\delta_{k}\left(t-\tau_{k}\right)I_{\tau_{k}}\left(t\right). \end{array}$$

When ${\tau_{k}}^{\prime }s$ are given, Model (2) and Model (3) are similar to regular linear and logistic regression, except that the model fitting is done by segments, as indicated by the additional covariates formed from the time intervals.

For a given data set, we are interested in answering the following questions: (1) Is (are) there any joinpoint(s)? If so, then (2) How many joinpoints (or change‐points) are there? (3) Where is (are) the joinpoint(s) located? and (4) How reliable are the parameter estimates? These are the same questions that the aggregate‐level modeling addresses. The next two sections lay out the proposed steps to answer these questions.

### 3.2 Model Selection for Given k(k>0)

For a given number of joinpoints k(k=1,2,…,K), we propose a grid search to find the best model (denoted by jpk) across models with different joinpoint locations $\tau_{1}$, …, $\tau_{k}$.

To identify model with the best joinpoint location(s), the grid search tests all possible joinpoint locations, following the same default rules as those implemented in the NCI Joinpoint software for aggregate‐level models [10]. The maximum number of joinpoints for the grid search is determined by the number of time points [11]. For more details, see the Supporting Information Materials.

For each possible model with given joinpoint(s) the following can be computed: $R^{2}$ for the log‐normal model (continuous data), and *−*2Log*L*, where logL is the log‐likelihood for the logistic model (binary data). The model with the largest $R^{2}$ or the smallest *−*2Log*L* will be chosen as the best model.

$R^{2}=1-\frac{{\mathrm{SS}}_{\text{error}}}{{\mathrm{SS}}_{\text{total}}}$, where

$${\mathrm{SS}}_{\text{error}}=\sum_{i=1}^{I}\sum_{t=1}^{T}\sum_{j=1}^{J_{i}^{\left(t\right)}}\sum_{l=1}^{n_{\mathrm{ij}}^{\left(t\right)}}w_{\mathrm{ijl}}^{\left(t\right)}{\left[\log\left(y_{\mathrm{ijl}}^{\left(t\right)}\right)-\hat{\log\left(y_{\mathrm{ijl}}^{\left(t\right)}\right)}\right]}^{2},$$

$${\mathrm{SS}}_{\text{total}}=\sum_{i=1}^{I}\sum_{t=1}^{T}\sum_{j=1}^{J_{i}^{\left(t\right)}}\sum_{l=1}^{n_{\mathrm{ij}}^{\left(t\right)}}w_{\mathrm{ijl}}^{\left(t\right)}{\left[\log\left(y_{\mathrm{ijl}}^{\left(t\right)}\right)-{\log\_\bar{y}}_{\mathrm{iw}}^{\left(t\right)}\right]}^{2},$$

$\hat{\log\left(y_{\mathrm{ijl}}^{\left(t\right)}\right)}$ is the predicted value of $\log\left(y_{\mathrm{ijl}}^{\left(t\right)}\right)$ and $\log\_{\bar{y}}_{\mathrm{iw}}^{\left(t\right)}=\frac{\sum_{j=1}^{J_{i}^{\left(t\right)}}\sum_{l=1}^{n_{\mathrm{ij}}^{\left(t\right)}}w_{\mathrm{ijl}}^{\left(t\right)}\log\left(y_{\mathrm{ijl}}^{\left(t\right)}\right)}{\sum_{j=1}^{J_{i}^{\left(t\right)}}\sum_{l=1}^{n_{\mathrm{ij}}^{\left(t\right)}}w_{\mathrm{ijl}}^{\left(t\right)}}$ is the weighted mean of $\log\left(y_{\mathrm{ijl}}^{\left(t\right)}\right)$ for stratum *i* at given *t*.

(4) $$\begin{array}{cc} -2\mathrm{LOG}L & =-2\sum_{i=1}^{I}\sum_{j=1}^{J_{i}}\sum_{l=1}^{n_{\mathrm{ij}}}w_{\mathrm{ijl}}^{\left(t\right)}\\ & \;\times \left[y_{\mathrm{ijl}}^{\left(t\right)}\log\left({\hat{\pi }}_{\mathrm{ijl}}^{\left(t\right)}\right)+\left(1-y_{\mathrm{ijl}}^{\left(t\right)}\right)\log\left(1-{\hat{\pi }}_{\mathrm{ijl}}^{\left(t\right)}\right)\right], \end{array}$$

where ${\hat{\pi }}_{\mathrm{ijl}}$ is the predicted probability of the event occurring for observation *l* in cluster *j* of stratum *i*.

### 3.3 Selecting the Best Model Using Modified dAIC

The grid search procedure selects the best jpk model for each given k(k=1,…,K). The next step is to select the best model from the candidate models jp*0*, jp*1*, jp*2*, …, jpK. A model selection criterion such as Akaike information criterion (AIC) is widely used in applied statistics. However, the classical AIC assumes the data had been selected through a simple random sample and thus cannot be applied directly to data that is collected via a complex survey sample design such as stratified multistage cluster sampling with potential correlation among observations from the same sampled cluster and varying sample weights. It also does not incorporate the non‐linear nature of a joinpoint model. Lumley and Scott [12] proposed a modified AIC (denoted as *dAIC*) to account for survey design in linear regression models with complex survey data (see Supporting Information Materials for details on *dAIC*). However, the *dAIC* cannot be applied directly to joinpoint models (2) and (3) due to the nonlinear nature with additional unknown parameters $\tau_{s},s=1,\ldots ,k.$

To address this issue, we propose a modified (m.dAIC), for selecting joinpoint models with survey data under complex sample designs. Let $\hat{\tau }=\left({\hat{\tau }}_{1},\ldots ,{\hat{\tau }}_{k}\right)$ denote the k estimated joinpoint locations for a given k (k=1,2,…,K) from the grid search. We propose the following m.dAIC:

For the log‐normal joinpoint model (2),

(5) $$m.\text{dAIC}=n\times \mathrm{MSE}+2\left(2k+1\right)\hat{\bar{\delta }};$$

and for the logistic joinpoint model (3),

(6) $$m.\text{dAIC}=\frac{n}{N}\times \left(-2\log L\right)+2\left(2k+1\right)\hat{\bar{\delta }},$$

where

(7) $$\mathrm{MSE}=\frac{\sum_{i=1}^{I}\sum_{t=1}^{T}\sum_{j=1}^{J_{i}^{\left(t\right)}}\sum_{l=1}^{n_{\mathrm{ij}}^{\left(t\right)}}w_{\mathrm{ijl}}^{\left(t\right)}{\left[\hat{\log\left(y_{\mathrm{ijl}}^{\left(t\right)}\right)}-\log\left(y_{\mathrm{ijl}}^{\left(t\right)}\right)\right]}^{2}}{\sum_{i=1}^{I}\sum_{t=1}^{T}\sum_{j=1}^{J_{i}^{\left(t\right)}}\sum_{l=1}^{n_{\mathrm{ij}}^{\left(t\right)}}w_{\mathrm{ijl}}^{\left(t\right)}};$$

−2logL is defined in formula (4) in Section 3.2.

$\hat{\bar{\delta }}$ is an estimated average design effect which is defined as: $\hat{\bar{\delta }}=\text{trace}\left\{\hat{J}{\left(\hat{\theta }\right)}^{-1}\hat{V}\left(\hat{\theta }\right)\right\},$ where $\hat{\theta }={\left({\hat{\beta }}_{0},{\hat{\beta }}_{1},{\hat{\delta }}_{1},\ldots ,{\hat{\delta }}_{k}\right)}^{\prime },$ $\hat{V}\left(\hat{\theta }\right)$ and $\hat{J}\left(\hat{\theta }\right)$ are estimated robust and model‐based variance–covariance matrix of $\hat{\theta },$ respectively. These can be derived using the same approach as that of generalized linear models for given $\hat{\tau }$ (for a detailed definition and derivation, refer to section 3 of the Supporting Information Material); 2k+1 is the number of unknown parameters in the joinpoint models (2) and (3) excluding the intercept parameter.

Since $\;\hat{\bar{\delta }}$ depends on the standard error estimates of the estimated regression coefficients, and thus we wanted to see how the accuracy of the model selection measure depends on these standard error estimates. In standard aggregate‐level joinpoint regression analysis, the covariance of the estimated regression coefficients is estimated using unconstrained model approach, for example, see [13, 14, 15]. As will be shown in our simulation studies, however, the performance of the model selection measure based on unconstrained standard error estimates was not satisfactory, and thus we also considered the average design effect estimate based on constrained model standard error estimates, which appeared to have better accuracy than the unconstrained model approach.

Therefore, to estimate$\;\hat{\bar{\delta }}$, we propose two approaches, which involve two methods of estimating the standard errors of the estimated regression coefficients:

1. Constrained model approach: Assume the mean functions are continuous at ${\hat{\tau }}_{s},\;s=1,\ldots ,k$.

For this approach, we fit the standard parameterized joinpoint regression model (2) for continuous outcome and model (3) for binary outcome, but replacing ($\tau_{1},\ldots ,\tau_{k})$ by (${\hat{\tau }}_{1},\ldots ,{\hat{\tau }}_{k})$ selected through the grid search.

2 Unconstrained model approach: Do not assume that the mean functions are continuous at ${\hat{\tau }}_{s}$.

For the second approach, we fit the following (k+1)‐phase (general parameterized) regression model without assuming that the mean functions are continuous at the ${\hat{\tau }}_{s}$:

For continuous outcome:

$$\log\left(y_{\mathrm{ijl}}^{\left(t\right)}\right)=\left(\beta_{1,0}+\beta_{1,1}t\right){\tilde{I}}_{\left[\min\left(t\right),{\hat{\tau }}_{1}\right)}\left(t\right)+\left(\beta_{2,0}+\beta_{2,1}t\right){\tilde{I}}_{\left({\hat{\tau }}_{1},{\hat{\tau }}_{2}\right)}\left(t\right)+$$

(8) $$\;+\left(\beta_{k+1,0}+\beta_{k+1,1}t\right){\tilde{I}}_{\left({\hat{\tau }}_{k},\max\left(t\right)\right]}\left(t\right)+\varepsilon_{\mathrm{ijl}}^{\left(t\right)};$$

For binary outcome:

(9) $$\begin{array}{cc} \text{logit}\left(p\left(y_{\mathrm{ijl}}^{\left(t\right)}=1\right)\right) & =\left(\beta_{1,0}+\beta_{1,1}t\right){\tilde{I}}_{\left[\min\left(t\right),{\hat{\tau }}_{1}\right)}\left(t\right)\\ & \;+\left(\beta_{2,0}+\beta_{2,1}t\right){\tilde{I}}_{\left({\hat{\tau }}_{1},{\hat{\tau }}_{2}\right)}\left(t\right)+\cdots \\ & \;+\left(\beta_{k+1,0}+\beta_{k+1,1}t\right){\tilde{I}}_{\left({\hat{\tau }}_{k},\max\left(t\right)\right]}\left(t\right); \end{array}$$

where $\beta_{s,0}$ and $\beta_{s,1}$are the intercept and slope of each segment line, s=1,…,k+1; ${\tilde{I}}_{B_{s}}\left(t\right)$ are the k+1 indicator variables defined as:

$${\tilde{I}}_{B_{s}}\left(t\right)=\left\{\begin{array}{l} 1,\;\text{if}\;t\in B_{s}=\left({\hat{\tau }}_{s-1},{\hat{\tau }}_{s}\right)\\ 0,\;\text{otherwise} \end{array}\right.$$

where ${\hat{\tau }}_{0}=\min\left(t\right)-1$ and ${\hat{\tau }}_{k+1}=\max\left(t\right)+1$, or we may define $B_{1}=\left[\min\left(t\right),{\hat{\tau }}_{1}\right)$ and $B_{k+1}=\left({\hat{\tau }}_{k},\max\left(t\right)\right]$. Note that for this approach, the offending observations (those that are the same as ${\hat{\tau }}_{s}$) are deleted in the modeling fitting.

The following steps are proposed (adapted from a similar approach used in conventional joinpoint regression models):

*Step* 1: Fit the unconstrained model (8) for continuous outcome and model (9) for binary outcome.

Let ${\hat{\beta }}_{k}={\left({\hat{\beta }}_{1,0},{\hat{\beta }}_{1,1},{\hat{\beta }}_{2,0},{\hat{\beta }}_{2,1},\ldots ,{\hat{\beta }}_{k+1,0},{\hat{\beta }}_{k+1,1}\right)}^{\prime }$ denote the 2(k+1)‐dimensional vector of the estimated regression coefficients for the unconstrained model. The estimated covariance matrices $\hat{J}\left({\hat{\beta }}_{k}\right)$ and $\hat{V}\left({\hat{\beta }}_{k}\right)$ are 2(k+2)×2(k+2) dimensions.

*Step* 2: Modify $\hat{J}\left({\hat{\beta }}_{k}\right)$ and $\hat{V}\left({\hat{\beta }}_{k}\right)$ as follows:

$$\hat{\tilde{J}}\left({\hat{\beta }}_{k}\right)=A_{k}\hat{J}\left({\hat{\beta }}_{k}\right)A_{k}^{\prime },\text{and}\hat{\tilde{V}}\left({\hat{\beta }}_{k}\right)=A_{k}\hat{V}\left({\hat{\beta }}_{k}\right)A_{k}^{\prime },$$

where $A_{k}$ is the (k+2)×(2k+2) matrix defined as in the Appendix and thus $\hat{\tilde{J}}$ and $\hat{\tilde{V}}$ are (k+2)×(k+2) dimensions. $A_{k}s$ are used to obtain the covariance matrix of the estimated regression coefficients for the constrained joinpoint model based on the covariance matrix estimated from the unconstrained model, which has been justified in literature for conventional joinpoint regression. The covariance matrix estimated from the constrained model conditional on the estimated locations of joinpoints does not appropriately incorporate uncertainty involved in the estimation of joinpoint locations, and thus it tends to underestimate the standard errors of the estimated regression coefficients. See Hinkley [16], Feder [13], and Kim and Kim [17] for theoretical justifications; Lerman [18] for practical implementations; and Kim and Kim [19] for empirical studies. The definition of $A_{k}$ can be found in the Appendix.

*Step* 3: Remove the first row and column of $\hat{\tilde{J}}$ and $\hat{\tilde{V}}$ so they are (k+1)×(k+1) dimensions.

*Step* 4: Compute $\hat{\bar{\delta }}$ as the trace of ${\hat{\tilde{J}}}^{-1}\hat{\tilde{V}}.$

As shown above, the difference between dAIC and our modified dAIC (m.dAIC) lies in two key aspects: (1) the modification of the number of parameters in the penalty term of the dAIC definition, and (2) the estimation of $\hat{\bar{\delta }}.$

### 3.4 Computation of APC and AAPC

Once the number and location of the joinpoints are identified, the APC and AAPC can be computed using a similar approach as described in Section 2 for the aggregate‐level analyses.

For the log‐normal joinpoint model (2), the model for the *s*th segment, s=1,…,k+1, is assumed as: $\log\left(y_{\mathrm{ijl}}^{\left(t\right)}\right)=\beta_{s,0}+\beta_{s,1}t+\varepsilon_{\mathrm{ijl}}^{\left(t\right)},$ where $\beta_{s,0}$ and $\beta_{s,1}$ denote the intercept and slope of the segment s, respectively, as defined in Section 2 for the aggregate case; also see Equations (8) and (9).

The estimated APC for the mean of outcome y (denoted as $\bar{y}$) from year t to year t+1 can also be derived as:

$$\begin{array}{cc} {\mathrm{APC}}_{s} & =100\left[{\hat{\bar{y}}}^{\left(t+1\right)}-{\hat{\bar{y}}}^{\left(t\right)}\right]/{\hat{\bar{y}}}^{\left(t\right)}\\ & \;=100\frac{\begin{array}{c} \sum_{i}\sum_{j}\sum_{l}w_{\mathrm{ijl}}{\hat{y}}_{\mathrm{ijl}}^{\left(t+1\right)}/\sum_{i}\sum_{j}\sum_{l}w_{\mathrm{ijl}}\\ \;-\sum_{i}\sum_{j}\sum_{l}w_{\mathrm{ijl}}{\hat{y}}_{\mathrm{ijl}}^{\left(t\right)}/\sum_{i}\sum_{j}\sum_{l}w_{\mathrm{ijl}} \end{array}}{\sum_{i}\sum_{j}\sum_{l}w_{\mathrm{ijl}}{\hat{y}}_{\mathrm{ijl}}^{\left(t\right)}/\sum_{i}\sum_{j}\sum_{l}w_{\mathrm{ijl}}}\\ & \;=100\left[e^{{\hat{\beta }}_{s,0}+{\hat{\beta }}_{s,1}\left(t+1\right)}-e^{{\hat{\beta }}_{s,0}+{\hat{\beta }}_{s,1}t}\right]/e^{{\hat{\beta }}_{s,0}+{\hat{\beta }}_{s,1}t}=100\left(e^{{\hat{\beta }}_{s,1}}-1\right), \end{array}$$

where ${\hat{\beta }}_{s,1}$ is the estimates of $\beta_{s,1}$ derived from the constrained model.

The corresponding 95% confidence interval of ${\mathrm{APC}}_{s}$ using parametric method is:

$$\left[100\left(e^{{\hat{\beta }}_{s,1}-\varphi }-1\right),100\left(e^{{\hat{\beta }}_{s,1}+\varphi }-1\right)\right],$$

where $\varphi =\mathrm{se}\left({\hat{\beta }}_{s,1}\right)\times c_{d}^{-1}\left(0.975\right)$, $\mathrm{se}\left({\hat{\beta }}_{s,1}\right)$ is the standard error of ${\hat{\beta }}_{s,1}$ and $c_{d}^{-1}\left(0.975\right)$ is the 97.5th percentile of the ***t***‐distribution with d degrees of freedom.

We use the degrees of freedom accounted for the complex survey design here, which equals to the total number of sampled PSUs minus the total number of strata. For large d, the interval could be a z‐interval. Like the aggregate‐level model approach, the standard error $\mathrm{se}\left({\hat{\beta }}_{s,1}\right)$ is estimated by fitting the unconstrained log‐normal linear model with the chosen joinpoints.

Similarly, the AAPC is estimated as:

$$\text{AAPC}=100\left[\exp\left(\frac{\sum \gamma_{s}{\hat{\beta }}_{s,1}}{\sum \gamma_{s}}\right)-1\right],$$

and the 95% confidence interval bounds are:

$$\begin{array}{cc} {\text{AAPC}}_{\text{Lper}} & =100\left\{\exp\left[\log\left(\left(\text{AAPC}/100\right)+1\right)\right.\right.\\ & \;\left.\left.-z_{0.975}\sqrt{{\tilde{\gamma }}^{\prime }\hat{V}\left(\hat{\beta }\right)\tilde{\gamma }}\right]-1\right\}, \end{array}$$

$$\begin{array}{cc} {\text{AAPC}}_{\text{Uper}} & =100\left\{\exp\left[\log\left(\left(\text{AAPC}/100\right)+1\right)\right.\right.\\ & \;\left.\left.+z_{0.975}\sqrt{{\tilde{\gamma }}^{\prime }\hat{V}\left(\hat{\beta }\right)\tilde{\gamma }}\right]-1\right\}, \end{array}$$

where $\hat{V}\left(\hat{\beta }\right)$ is the estimated variance of $\hat{\beta }=\left({\hat{\beta }}_{1,1},\ldots ,{\hat{\beta }}_{k+1,1}\right)$ derived from the unconstrained model, ${\tilde{\gamma }}_{s}=\gamma_{s}/\sum \gamma_{s},$ and $\gamma_{s}$ equals to the length of the APC interval s,s=1,…,k+1.

For the logistic joinpoint model (3), let ${\text{Odds}}_{y_{\mathrm{ijl}}^{\left(t\right)}=1}=\frac{\text{prob}\left(y_{\mathrm{ijl}}^{\left(t\right)}=1\right)}{1-\text{prob}\left(y_{\mathrm{ijl}}^{\left(t\right)}=1\right)},$ the model for the sth segment is assumed as: $\log\left({\text{Odds}}_{y_{\mathrm{ijl}}^{\left(t\right)}=1}\right)=\beta_{s,0}+\beta_{s,1}t,$ where $\beta_{s,0}$ and $\beta_{s,1}$ denote the intercept and slope of the segment s, respectively. The estimated APC for the odds, that is, sample weighted mean of the predicted odds for each individual at a given time, of the outcome y from year t to year t+1, denoted as OAPC, can also be derived as:

$$\begin{array}{cc} {\text{OAPC}}_{s} & =100\frac{\begin{array}{c} \sum_{i}\sum_{j}\sum_{l}w_{\mathrm{ijl}}{\hat{\text{Odds}}}_{y_{\mathrm{ijl}}^{\left(t+1\right)}=1}/\sum_{i}\sum_{j}\sum_{l}w_{\mathrm{ijl}}\\ \;-\sum_{i}\sum_{j}\sum_{l}w_{\mathrm{ijl}}{\hat{\text{Odds}}}_{y_{\mathrm{ijl}}^{\left(t\right)}=1}/\sum_{i}\sum_{j}\sum_{l}w_{\mathrm{ijl}} \end{array}}{\sum_{i}\sum_{j}\sum_{l}w_{\mathrm{ijl}}{\hat{\text{Odds}}}_{y_{\mathrm{ij}l}^{\left(t\right)}=1}/\sum_{i}\sum_{j}\sum_{l}w_{\mathrm{ijl}}}\\ & \;=100\left(e^{{\hat{\beta }}_{s,1}}-1\right), \end{array}$$

where ${\hat{\beta }}_{s,1}$ is the estimates of $\beta_{s,1}.$ The corresponding 95% confidence interval of $O{\mathrm{APC}}_{s}$ using parametric method is:

$$\left[100\left(e^{{\hat{\beta }}_{s,1}-\varphi }-1\right),100\left(e^{{\hat{\beta }}_{s,1}+\varphi }-1\right)\right],$$

where $\varphi =\mathrm{se}\left({\hat{\beta }}_{s,1}\right)\times c_{d}^{-1}\left(0.975\right)$. The standard error $\mathrm{se}\left({\hat{\beta }}_{s,1}\right)$ is estimated by fitting the unconstrained logistic model with the chosen joinpoints.

The AAPC of the odds of the outcome y, denoted as OAAPC, and the 95% confidence interval $({\text{OAAPC}}_{\text{Lper}},$ ${\text{OAAPC}}_{\text{Uper}})$ can be derived using the same form as the AAPC and $({\text{AAPC}}_{\text{Lper}},$ ${\text{AAPC}}_{\text{Uper}})$ for the continuous case, except that the parameters are estimated from the corresponding logistic joinpoint models.

### 3.5 Model Implementation

Appropriate survey analysis computer packages (e.g., the *svyglm* procedure in R, the SAS *PROC SURVEYREG*/*SURVEYLOGISTIC*, or the SUDAAN *PROC REGRESS/RLOGIST*) can be used to fit the unit‐level joinpoint models and compute the necessary statistics for a given number of joinpoint(s) and joinpoint location(s).

## 4 Simulation Studies

### 4.1 Data Generation

Our objective was to generate 20 years of data (*t* = 1997–2016) to evaluate the proposed joinpoint methods. Without loss of generality, we generated a finite target population (FTP) consisting of one million individuals, divided into 1000 equal‐sized clusters, with data for one continuous outcome and one binary outcome, each with one true joinpoint. The FTP data was generated using four sets of intra‐cluster correlation coefficient (*ICC*) values (0, 0.01, 0.075, 0.3), three APC difference values (0,−0.41%,−4.1%) for the continuous outcome, and four OAPC difference values (0,−0.485%,−4.85%,−28.5%) for the binary outcome.

For each year, 80 clusters were sampled from the 1000 FTP clusters. To mimic the National Health Interview Survey (NHIS), sampling of the FTP clusters overlapped across years: the first 10 years used the same set of sampled clusters, and the second 10 years used another set. To evaluate the effect of sample size, for each year and sampled cluster, an independent random sample of individuals were selected with pre‐defined sampling rates of 5%, 20%,50%, or a varying sampling rate (random drawn from a uniform distribution between 5% and 15%) for cases with APC differences of −4.1% or OAPC differences of −4.85% or −28.5%.

For each scenario, sampling was repeated 100 times to form 100 samples, each containing 20 years of data. Further details on data generation are provided in the Supporting Information Material.

The preset parameters for data generation were based on analyses of continuous body mass index and binary obesity data from NHIS. For cluster sampling, Kish's design effect, $1+\left(\bar{n}-1\right)\mathrm{ICC},$ depends on both *ICC* and average cluster size $\bar{n}$ [20]. Both the *ICC* and average cluster size may vary across surveys. For well‐designed surveys, the design effect typically ranges from 1 to 3, though it can be larger—up to 7 or 8, or even 30 [21], for example, for institutional surveys. In this simulation study, $\bar{n}$ ranged from 50 to 500, and ICC values were set up to 0.075, reflecting typical design effects in national household surveys. Larger ICC (0.3) and OAPC differences (−28.5%) were also tested to assess the proposed methodology. See Table S1 for design effect under each scenario.

### 4.2 Analyses of Results

With each set of sampled data for each scenario, we ran the individual‐level models and the aggregate‐level models with and without full variance–covariance matrix based on weighted BIC (WBIC) method [15] for both the continuous outcome and binary outcome generated. We compared how many times the number of joinpoints and the locations were correctly identified using the design factor estimates based on constrained and unconstrained individual‐level model standard error estimates and those from the aggregate‐level models. We also checked which estimator, the variance estimator $\hat{V}\left(\hat{\theta }\right)$ from the constrained model or the $\hat{\tilde{V}}$ from the unconstrained model, is a better estimator of the true covariance matrix. To do this, we did the following steps:

1. Retain the estimates of $\beta_{0}^{\left(b\right)}$, $\beta_{1}^{\left(b\right)},$ and $\delta_{1}^{\left(b\right)}$ and the three elements in the diagonal of the covariance matrix estimator $\hat{V}\left(\hat{\theta }\right)$ under constrained model and $\hat{\tilde{V}}$ under the unconstrained model (denoted as $C_{11}^{\left(b\right)}$, $C_{22}^{\left(b\right)},$ and $C_{33}^{\left(b\right)}$) from the jk1 model for each resampling of the FTP, b=1,…,100.
2. Calculate the empirical means and Monte Carlo errors (standard deviations) for each parameter were computed as follows:

The means over the 100 repeated sampling ${\hat{\bar{\beta }}}_{0}=\sum_{b=1}^{100}{\hat{\beta }}_{0}^{\left(b\right)}/100,$ ${\hat{\bar{\beta }}}_{1}=\sum_{b=1}^{100}{\hat{\beta }}_{1}^{\left(b\right)}/100,$ ${\hat{\bar{\delta }}}_{1}=\sum_{b=1}^{100}{\hat{\delta }}_{1}^{\left(b\right)}/100$, and the standard deviations as

$$\mathrm{SD}\left({\hat{\bar{\beta }}}_{0}\right)=\sqrt{\sum_{b=1}^{100}{\left({\hat{\beta }}_{0}^{\left(b\right)}-{\hat{\bar{\beta }}}_{0}\right)}^{2}/\left(100-1\right)},$$

$$\mathrm{SD}\left({\hat{\bar{\beta }}}_{1}\right)=\sqrt{\sum_{b=1}^{100}{\left({\hat{\beta }}_{1}^{\left(b\right)}-{\hat{\bar{\beta }}}_{1}\right)}^{2}/\left(100-1\right)},$$

$$\mathrm{SD}\left({\hat{\bar{\delta }}}_{1}\right)=\sqrt{\sum_{b=1}^{100}{\left({\hat{\delta }}_{1}^{\left(b\right)}-{\hat{\bar{\delta }}}_{1}\right)}^{2}/\left(100-1\right)}.$$

3 Compute the corresponding empirical variances ${\hat{\bar{C}}}_{11},{\hat{\bar{C}}}_{22}$, and ${\hat{\bar{C}}}_{33}$ for the constrained and unconstrained models corresponding to diagonal terms in the variance–covariance matrices, respectively: ${\hat{\bar{C}}}_{11}=\sum_{b=1}^{100}{\hat{C}}_{11}^{\left(b\right)}/100,{\hat{\bar{C}}}_{22}=\sum_{b=1}^{100}{\hat{C}}_{22}^{\left(b\right)}/100,$ and ${\hat{\bar{C}}}_{33}=\sum_{b=1}^{100}{\hat{C}}_{33}^{\left(b\right)}/100.$

### 4.3 Simulation Results

Table 1 presents the percentage (%) of repeated samples from simulation that correctly identified the true number of joinpoints for each scenario with *ICC* ≤ 0.075 from the two aggregate‐level approaches and the two individual‐level approaches for the continuous outcome case. The results indicate that the percentage of correct identifications generally increases as the APC difference increases, regardless of the sampling rate. For the constrained individual‐level models, correct identification increased as ICC increased, reaching ≥ 95% when ICC > 0. When the ICC = 0, rates ranged from 86% to 93%. For the unconstrained individual‐level models, correct identification rates decreased as ICC increased, with the best rates ranged 59% to 90% for ICC = 0 and rates mostly < 50% for ICC > 0. When ICC = 0, aggregate‐level models outperformed constrained individual‐level models. The aggregate‐level models with covariance adjustment performed better than those without when ICC > 0, confirming the results of Liu et al. [7]. However, for ICC > 0, constrained individual‐level models performed better, likely due to the individual‐level model's more accurate handling of design information.

**TABLE 1:** Percent (%) of samples correctly identified true number of joinpoint(s) for a continuous outcome: Log‐normal models.

Sampling rate	APC difference	ICC = 0				ICC = 0.01				ICC = 0.075			
		Aggregate‐level model		Individual‐level model		Aggregate‐level model		Individual‐level model		Aggregate‐level model		Individual‐level model	
		Without COV	With COV	Constrained	Unconstrained	Without COV	With COV	Constrained	Unconstrained	Without COV	With COV	Constrained	Unconstrained
5% (*n* = 4000)	0.00%	***93***	89	90	76	94	88	***97***	30	83	89	***100***	0
	−0.41%	***94***	***94***	90	67	91	***95***	***95***	36	85	93	***95***	28
	−4.10%	***97***	96	93	78	94	***97***	96	51	83	96	***98***	21
20%	0.00%	***91***	88	86	77	86	90	***100***	0	53	87	***100***	0
	−0.41%	***94***	93	89	59	84	95	***98***	22	58	94	***99***	22
	−4.10%	95	***96***	90	71	91	96	***99***	22	61	96	***100***	14
50%	0.00%	***91***	89	87	78	72	88	***100***	0	40	88	***100***	0
	−0.41%	***94***	***94***	87	69	71	91	***97***	20	51	92	***99***	14
	−4.10%	***95***	***95***	89	72	73	92	***99***	23	73	92	***98***	10
uniform 5% ˜ 15%	−4.10%	94	***100***	91	90	91	***100***	96	45	75	***100***	97	16

*Note:* The highest value within each scenario is bold and italicized.

Correct identification rates for each scenario with *ICC* = 0.3 are presented in Table S2. Aggregate‐level model with covariance adjustment achieved ≥ 90% accuracy for all scenarios, while constrained individual‐level model approach achieved ≥ 98% for all scenarios except those with non‐zero but very small APC difference (−0.41%). In cases when APC difference was −0.41%, most runs using constrained individual‐level model approach selected zero‐joinpoint model, likely due to insufficient power caused by the large design effect. Both aggregate‐level model without covariance adjustment and unconstrained individual‐level model approach performed poorly in these scenarios.

Table 2 shows the correct identification rates for models with the binary outcome across scenarios with *ICC* ≤ 0.075. These rates are generally lower than those from the continuous outcome and vary by OAPC difference. For relatively larger OAPC differences (−4.85%), correct identification rates ranged from 69% to 100% across model methods and data scenarios. Overall, constrained individual‐level models slightly outperformed unconstrained ones, while aggregate‐level models generally achieved higher rates. However, the difference between the two model types diminished with larger ICCs and APCs. The few extremely low rates were likely due to the non‐linear property of logistic models.

**TABLE 2:** Percent (%) of replicates correctly identify true number of joinpoint(s) for a binary outcome: Log‐normal for aggregate‐level proportion and individual‐level logistic models.

Sampling rate	OAPC difference	ICC = 0				ICC = 0.01				ICC = 0.075			
		Aggregate‐level model		Individual‐level model		Aggregate‐level model		Individual‐level model		Aggregate‐level model		Individual‐level model	
		Without COV	With COV	Constrained	Unconstrained	Without COV	With COV	Constrained	Unconstrained	Without COV	With COV	Constrained	Unconstrained
5% (*n* = 4000)	0	69	***73***	72	71	***74***	***74***	71	71	***76***	74	*75*	*75*
	−0.485%	38	35	18	21	36	***38***	26	24	32	35	32	31
	−4.85%	***88***	87	72	69	***86***	82	73	74	***85***	83	83	83
20% (*n* = 16 000)	0	31	24	74	75	***64***	60	82	84	71	70	75	***77***
	−0.485%	***86***	76	42	38	60	***62***	35	34	37	39	33	37
	−4.85%	***90***	89	74	73	***85***	82	76	73	***93***	88	92	87
50% (*n* = 40 000)	0	3	7	73	76	55	53	***84***	***84***	72	70	**73**	69
	−0.485%	***91***	84	57	58	***70***	***70***	34	33	37	**38**	34	36
	−4.85%	***91***	90	76	74	91	***92***	84	78	**92**	88	91	88
Uniform 5% ˜ 15% (average *n* = 8000)	−4.85%	93	***100***	74	73	92	***100***	72	78	86	80	81	***87***

*Note:* The highest value within each scenario is bold and italicized.

Table S3 presents correct identification percentages for scenarios with *ICC* = 0.3. Performance varied with OAPC differences. For a large OAPC difference (−24.25%), the aggregate‐level model (with or without covariance adjustment) achieved ≥ 93% correct identification, with the covariance‐adjusted model reaching 100% in three of four sampling rate scenarios. Individual‐level models achieved 77%–82%. When the OAPC difference was zero, all models achieved ≥ 80% correct identification (up to 91%), with the covariance‐adjusted aggregate‐level model performing the best in most scenarios. For small but non‐zero OAPC differences (−0.485% or −4.85%), no model performed well (≤ 52%), likely due to insufficient power from the large design effects.

**FIGURE 1:**
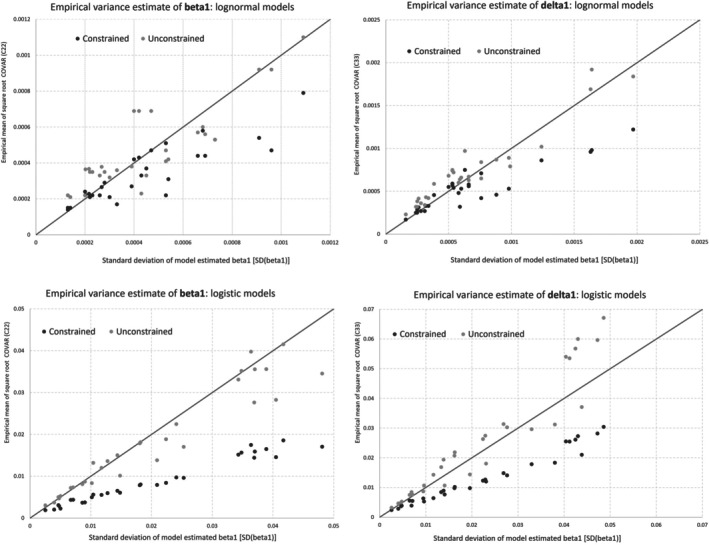
Variance estimation of beta 1 and delta 1 from the individual‐level models.

Figure 1 presents the variance estimation of ${\hat{\beta }}_{1}$ and ${\hat{\delta }}_{1}$ based on both the constrained approach and unconstrained approach (i.e., square root of $\;{\hat{\bar{C}}}_{22}$, ${\hat{\bar{C}}}_{33}$) versus the empirical standard deviation $\mathrm{SD}\left({\hat{\bar{\beta }}}_{1}\right)$ and $\mathrm{SD}\left({\hat{\bar{\delta }}}_{1}\right)$ for the lognormal and logit models, respectively. The figures illustrate that the standard errors from the unconstrained models scatter around the diagonal line, whereas those from the constrained model are not as closely aligned and tend to fall below it. This indicates that, in comparison to the empirical standard errors from the simulations, the estimation of the standard errors from the unconstrained models performs well, while the constrained modeling standard errors are more biased and tend to underestimate the true standard errors.

As we mentioned in Section 3.3, in conventional joinpoint regression, the use of standard error estimates based on the unconstrained model is both theoretically and empirically established. It has been theoretically proved that the asymptotic variance of the estimated regression coefficients should be based on the unconstrained model, as empirical studies show that standard error estimates from the constrained model tend to underestimate variability by not accounting for joinpoint location variability [13, 16, 17, 18, 19]. Based on our simulation results, we recommend using constrained modeling approach to compute m.dAIC and determine the number and location of joinpoints of the final model when the design effect is reasonable. Once the final joinpoint model is selected, the point estimates of APC, OAPC, AAPC, and OAAPC should be derived from the slope estimates of the constrained models. However, the standard errors for these estimates should come from the corresponding unconstrained model to compute the 95% confidence intervals of APC (OAPC) and AAPC (OAAPC) as described in Section 3.5. This approach aligns with the methodology used in NCI's Joinpoint for APC and AAPC computations, which rely on standard aggregate‐level joinpoint models.

## 5 Empirical Study

### 5.1 Data Sources and Measures

For this empirical study, we use data from the NHIS, an annual cross‐sectional household survey utilizing a stratified multi‐stage cluster (geographically based clusters) probability sample design that is a representative random sample of the US households and noninstitutional group quarters. The same sampled PSUs are used for each year between redesign of the samples, which occurs generally after every decennial census.

We extracted the NHIS 1991–2016 data that included variables body mass index (BMI, defined as the body weight in kilograms divided by the square of the body height in meters), year, age, survey weight, and design information (codes for stratum and PSU) from the NHIS IPUMS website (https://nhis.ipums.org/nhis/). The analysis was restricted to adult respondents aged 18 and over whose BMI was less than 99.9 due to questionable validity (*n* = 1 005 847). A binary obesity variable was defined as obesity = 1 if BMI ≥ 30; obesity = 0, otherwise.

### 5.2 Statistical Analysis

Using the individual‐level data, we fit a log‐normal joinpoint model (2) for continuous BMI and a logistic joinpoint model (3) for obesity, did a grid search to find the best joinpoint models for each number of joinpoints *k*, *k* = 1, …, 4 (denoted as jp*k*), estimated $\hat{\bar{\delta }}$ using both constrained and unconstrained models, computed m.dAIC for each of the best jp*k* models and jp0 models, that is, with no joinpoints, and finally selected the final model with the minimum m.dAIC.

For comparison purposes, we conducted an aggregate‐level analysis. We first computed the yearly mean BMI and yearly proportion of obesity using a survey analysis computer package (e.g., SAS or R) to incorporate the survey weights and design information. We then fit the aggregate level model (1) with and without incorporating the full variance–covariance matrix of the estimated parameters $\theta ={\left(\beta_{0},\beta_{1},\delta_{1},\ldots ,\delta_{k}\right)}^{\prime }$ using the NCI Joinpoint software to select the best model and estimate the parameters.

### 5.3 Results

**FIGURE 2:**
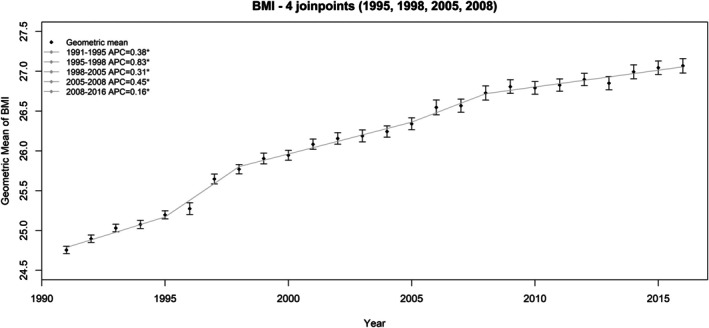
Plot of the yearly geometric mean along with the 95% confidence intervals and the fitted joinpoint line from individual‐level log‐normal model for BMI chosen from the constrained approach (with four joinpoints: 1995, 1998, 2005, 2008. *The APC is significant at alpha = 0.05).

**FIGURE 3:**
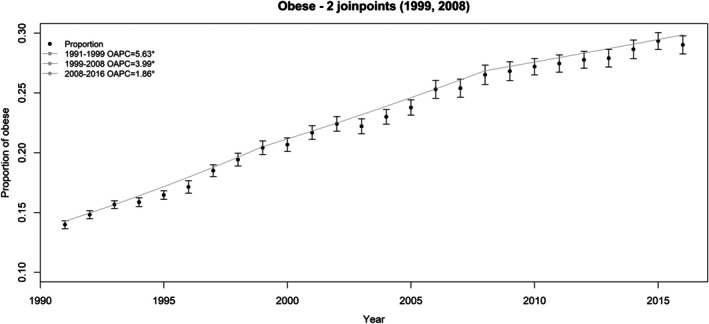
Plot of the yearly proportion of obese (and 95% confidence interval) along with the fitted joinpoint line estimated from the individual‐ level logistic model for obese chosen from the constrained approach (with two joinpoints: 1999 and 2008; the OAPC is for the annual percent change of the odd of being obese. *The OAPC is significant at alpha = 0.05).

Figure 2 displays the BMI data, showing the annual geometric mean of BMI with 95% confidence intervals, overlaid with the fitted joinpoint line estimated from the individual‐level log‐normal joinpoint model selected using the constrained approach. Similarly, Figure 3 shows the yearly proportion of obese with pointwise 95% confidence intervals, overlaid with the fitted joinpoint model line estimated from the unit‐level logistic model selected using the constrained approach.

Table 3 presents the final number of joinpoints and APC and AAPC results for the aggregate‐level and individual‐level log‐normal models for annual mean BMI estimates. The 95% CIs for both APC and AAPC were computed using *Z*‐intervals, as the denominator degree of freedom was large enough (= 1722). The aggregate‐level models, with or without covariance matrix, identified two joinpoints at the same locations (1999, 2009). In contrast, the individual‐level models selected four joinpoints (1995, 1998, 2005, and 2008). All APC and AAPC results were statistically significant (α=0.05). APC and AAPC results were nearly identical in the aggregate‐level models, with or without incorporating the covariance matrix. The AAPC estimates across all models were similar, and AAPCs for the time ranges 1991–1999, 1999–2009, and 2009–2016, computed using the individual‐level models, were not significantly different from the APCs from the aggregate‐level models. However, the confidence intervals from individual‐level models were generally wider, likely due to the incorporation of correct degrees of freedom and survey design information.

**TABLE 3:** The final joinpoints identified and the APC (%) and AAPC (%) results from the log‐normal models for mean BMI.

	Aggregate‐level model				Individual‐level model	
	Without COV		With COV		1995, 1998,	
Joinpoints	1999, 2009		1999, 2009		2005, 2008	
Segment	Range	APC (95% CI)	Range	APC (95% CI)	Range	APC (95% CI)
1	1991–1999	0.54 (0.48, 0.60)	1991–1999	0.54 (0.48, 0.59)	1991–1995	0.38 (0.32, 0.45)
2	1999–2009	0.37 (0.30, 0.43)	1999–2009	0.36 (0.29, 0.42)	1995–1998	0.83 (0.45, 1.21)
3	2009–2016	0.17 (0.07, 0.27)	2009–2016	0.18 (0.08, 0.28)	1998–2005	0.31 (0.24, 0.37)
4					2005–2008	0.45 (0.03, 0.87)
5					2008–2016	0.16 (0.11, 0.21)

**AAPC**						
Whole range	1991–2016	0.37 (0.33, 0.41)	1991–2016	0.37 (0.33, 0.41)	1991–2016	0.35 (0.28, 0.42)
Range corresponding to the aggregate‐level model					1991–1999	0.54 (0.39, 0.69)
					1999–2009	0.33 (0.20, 0.47)
					2009–2016	0.16 (0.11, 0.21)

*Note:* The final number and location of joinpoints from the individual‐level model were determined using the constrained model approach.

Table 4 presents the summary results from logistic joinpoint models for proportion of obese. The two aggregate‐level approaches, with or without the covariance matrix, produced very similar results. However, the individual‐level constrained and unconstrained approaches yielded different numbers of joinpoints. The constrained approach identified the same number and locations of joinpoints as the aggregate level approach (1999 and 2008), while the unconstrained approach identified more joinpoints (1996, 1999, 2004, and 2008). Only the results from the constrained model selection approach are presented in Table 4, as this is the recommended approach based on the simulation results.

**TABLE 4:** The final joinpoints identified and the OAPC (%) and OAAPC (%) results for obesity: Aggregate‐level log‐normal models and individual‐level logistic models.

	Aggregate level				Individual‐level	
	Without COV		With COV			
Joinpoints	1999, 2008		1999, 2008		1999, 2008	
Segment	Range	APC (95% CI)	Range	APC (95% CI)	Range	OAPC (95% CI)
1	1991–1999	4.62 (4.13, 5.11)	1991–1999	4.65 (4.17, 5.14)	1991–1999	5.63 (5.02, 6.20)
2	1999–2008	3.03 (2.49, 3.58)	1999–2008	3.00 (2.45, 3.45)	1999–2008	3.99 (3.35, 4.64)
3	2008–2016	1.35 (0.85, 1.85)	2008–2016	1.37 (0.87, 1.88)	2008–2016	1.86 (1.28, 2.45)

AAPC					OAAPC	
Whole range	1991–2016	2.99 (2.72, 3.27)	1991–2016	3.00 (2.72, 3.28)	1991–2016	3.82 (3.47, 4.17)

*Note:* The APC and AAPC from the aggregate level models are for the annual change of proportions of being obese; those from the individual‐level model are for the annual change of the average odd of being obese.

For the individual‐level logistic models, as described in Section 3.5, the definition of APC and AAPC differ from those in the log‐normal models and are denoted as OAPC and OAAPC. However, the magnitude of the OAPCs and OAAPCs from the individual‐level models align with the APCs and AAPC from the aggregate‐level models. The OAPCs from the individual‐level constrained model for the three segments (1991–1999, 1999–2008, and 2008–2016) are larger than those from the aggregate‐level model, but the differences are not significant due to overlapping 95% confidence intervals. In contrast, the OAAPC from the individual‐level model is significantly larger than the AAPCs from the aggregate‐level models for the 1991–2016 range (3.82 [3.49, 4.15] vs. 3.00 [2.72, 3.28] or 2.99 [2.72, 3.27]).

## 6 Summary and Discussion

In this paper, we propose individual‐level joinpoint methods for trend analysis from complex survey data within a finite population framework. These methods are evaluated using both simulated data and empirical data and compared with existing aggregate‐level methods. Performance is assessed across various scenarios, including different combinations of APC (OAPC) differences, ICCs, and sample sizes, focusing on designs typical of national household surveys. These methods are also applicable to institutional surveys, such as the US National Hospital Discharge Survey [22] and the National (Nationwide) Inpatient Sample [23].

The individual‐level constrained model outperforms aggregate‐level models, in identifying the correct number of joinpoints, especially for sample designs with higher ICCs and reasonable design effects. In these cases, the individual‐level approach takes advantage of the larger degrees of freedom for estimation of the design‐based variance–covariance matrix. Aggregate‐level models perform better for designs with zero or low ICCs where the larger number of degrees of freedom from the identity of the sampled PSU's do not contribute information to the estimation of the variances of the estimate regression coefficients $\hat{\theta }$. Our simulations suggest that the individual‐level constrained model approach is preferred for survey data with ICC > 0 and reasonable design effects, while aggregate‐level models are suitable for low ICC data.

For standard aggregate‐level joinpoint model (with non‐survey data), the AIC method tends to overestimate the model, selecting too many joinpoints due to small penalty [15]. In contrast, individual‐level joinpoint models show that constrained AIC standard error estimates tend to underestimate true variance of the estimated regression coefficients. Our simulations indicate that the unconstrained model provided better standard error estimates, but with worse joinpoint identification, likely due to the liberal nature of the AIC approach in aggregate‐level data as noted above.

For individual‐level joinpoint modeling, we recommend using the constrained modeling approach to determine the number and location of joinpoints. After selecting the final joinpoint model, compute APC (OAPC) and AAPC (OAAPC) using the constrained model's slope estimates and derive standard errors from the corresponding unconstrained model.

NCI's Joinpoint software was originally designed for time‐point estimates from super population (SP) framework, not finite population samples. Users of earlier versions of Joinpoint (prior to version 4.9) treated time‐point estimates from complex sample surveys or simple random samples selected from a FP as if they were only derived from a SP. SP inference could be integrated into FP inference by adding variability due to realizations from an SP to the variances of the FP inference (see Graubard and Korn [24] for an approximately distribution‐free SP framework), but this could require knowledge of non‐publicly available sample design information, for example, in national surveys such as the NHIS the identity of PSUs sample with large sampling fractions and joint inclusion probabilities of the sampled PSUs. Xu et al. [25] proposed a Bayesian information criterion (BIC) approach (*BIC*_*n*_) for variable selection in survey data under a simpler SP framework that does not account for the realizations of sampling strata and sample clustering in the FP that may be used in sample selection from the FP [24]. Lumley and Scott [12] proposed a design‐based version of BIC (*dBIC*) and demonstrated that *dBIC* equals *BIC*_*n*_ under certain conditions. We considered extending Lumley and Scott's *dBIC* approach to joinpoint model selection but decided to use the sample design‐based *dAIC* approach, as the maximal model required for *dBIC* does not exist for joinpoint models.

In our empirical study of the NHIS, APCs and AAPCs from individual‐level models generally aligned with aggregate‐level models, though sometimes different findings were noted. Occasionally the individual‐level joinpoint model detected joinpoints with very small APC (or OAPC) changes, which may not be meaningful. We recommend implementing a Minimum APC/OAPC Difference Worth Detecting (MADWD) for individual‐level analyses, similar to the algorithm in NCI's Joinpoint software for aggregate‐level model [26].

Individual‐level modeling offers the advantage of adjusting for individual‐level covariates that are associated with outcomes and vary over time. Although covariates inclusion was not explored here, it will be addressed in future research. The algorithms are developed in R, and portions of them have been validated by comparing results with those obtained using SAS and SUDAAN and will be released on the NCI's Joinpoint website upon the publication of this paper. Future versions of the software may include additional features to make this adaptation of the original Joinpoint methodology more easily accessible for a broader range of users.

Note that joinpoint regression, which employs a piecewise linear model with abrupt shifts, is typically used to identify the timing and significance of trend changes. Other model types, such as smoothly varying models or autoregressive Integrated moving average (ARIMA) models, serve different purposes and have distinct characteristics that may be better for addressing certain types of time series data, for example, interrupted time series as found in intervention studies [27]. These model types are beyond the scope of this paper.

## Funding

The authors have nothing to report.

## Conflicts of Interest

The authors declare no conflicts of interest.

## Supporting information


**Data S1:** sim70374‐sup‐0001‐Supinfo.docx.

## Data Availability

The data that support the findings of this study are openly available in the NHIS IPUMS website (https://nhis.ipums.org/nhis/).

## Appendix A

*The* $A_{k}$ *matrices*.

Here are the $A_{k}$ matrix, k=1,…,4, used in this study to modify the model‐based and robust variances of the unknown parameters when estimating $\hat{\bar{\delta }}$ for the modified *m.dAIC* computation.

$$A_{1}=\left(\begin{array}{ll} 1 & 0\\ 0 & 1\\ 0 & -1 \end{array}\;\begin{array}{ll} 0 & 0\\ 0 & 0\\ 0 & 1 \end{array}\right),\;A_{2}=\left(\;\begin{array}{ll} \begin{array}{lll} 1 & 0 & 0\\ 0 & 1 & 0 \end{array} & \begin{array}{lll} 0 & 0 & 0\\ 0 & 0 & 0 \end{array}\\ \begin{array}{lll} 0 & -1 & 0\\ 0 & 0 & 0 \end{array} & \begin{array}{lll} 1 & 0 & 0\\ -\;1 & 0 & 1 \end{array} \end{array}\right),$$

$$\;A_{3}=\left(\begin{array}{lll} \begin{array}{lll} 1 & 0 & 0\\ 0 & 1 & 0\\ 0 & -1 & 0 \end{array} & \begin{array}{lll} 0 & 0 & 0\\ 0 & 0 & 0\\ 1 & 0 & 0 \end{array} & \begin{array}{ll} 0 & 0\\ 0 & 0\\ 0 & 0 \end{array}\\ \begin{array}{lll} 0 & 0 & 0\\ 0 & 0 & 0 \end{array} & \begin{array}{lll} -\;1 & 0 & 1\\ 0 & 0 & -1 \end{array} & \begin{array}{ll} 0 & 0\\ 0 & 1 \end{array} \end{array}\right)$$

$$\;A_{4}=\left(\begin{array}{ll} \begin{array}{lll} 1 & \;0 & 0\\ 0 & \;1 & 0\\ 0 & -1 & 0 \end{array}\;\begin{array}{ll} 0 & 0\\ 0 & 0\\ 1 & 0 \end{array} & \begin{array}{ll} \begin{array}{lll} 0 & 0 & 0\\ 0 & 0 & 0\\ 0 & 0 & 0 \end{array} & \begin{array}{ll} 0 & 0\\ 0 & 0\\ 0 & 0 \end{array} \end{array}\\ \begin{array}{ll} \begin{array}{lll} 0 & 0 & 0\\ 0 & 0 & 0\\ 0 & 0 & 0 \end{array} & \begin{array}{ll} -\;1 & 0\\ 0 & 0\\ 0 & 0 \end{array} \end{array} & \begin{array}{ll} \begin{array}{lll} 1 & 0 & 0\\ -\;1 & 0 & 1\\ 0 & 0 & -1 \end{array} & \begin{array}{ll} 0 & 0\\ 0 & 0\\ 0 & 1 \end{array} \end{array} \end{array}\right)$$

$A_{k}s$ are used to obtain the covariance matrix of the estimated regression coefficients for the constrained joinpoint model based on the covariance matrix estimated from the unconstrained model, which has been justified in literature for conventional joinpoint regression [13, 16, 17, 18, 19]. When k>4, $A_{k}s$ can be derived using similar patterns as those for k≤4.
