# Supplementary material for: Extended Joinpoint Regression Methodology for Complex Survey Data
Source: Stat Med. 2026 Jan 22;45(1-2):e70374. doi: 10.1002/sim.70374 (PMC12828251; doi:10.1002/sim.70374)
Supplement: Supplementary file 1 — Data S1: sim70374‐sup‐0001‐Supinfo.docx. [file SIM-45-0-s001.docx]

**Supplementary materials for “****Extended Joinpoint Regression Methodology for Complex Survey Data”**

1. **The 95% CI of the** $\boldsymbol{APC}_{\boldsymbol{sA}}$ **and** $\boldsymbol{AAPC}_{\boldsymbol{A}}$

Using the same notations as given in Section 2 of the main text, the corresponding 95% confidence interval of the annual percent change ${(APC}_{sA})$ using the parametric method is:

$\left[ 100\left( e^{\hat{\beta}_{s,A1}-\varphi}-1 \right), 100\left( e^{\hat{\beta}_{s,A1}+\varphi}-1 \right) \right]$,

where $\varphi=se\left( \hat{\beta}_{s,A1} \right)\times c_{d}^{-1}\left( 0.975 \right)$, $se\left( \hat{\beta}_{s,A1} \right)$ is the standard error of $\hat{\beta}_{s,A1}$ and $c_{d}^{-1}\left( 0.975 \right)$ is the 97.5^th^ percentile of the ***t***-distribution with $d$ degrees of freedom, $d=T-\left( 2k+2 \right)$ when no offending observations, i.e., observations at the time points that coincide with the estimated joinpoints, are deleted; $d=T^{*}-\left( 2k+2 \right)$when the offending observations are deleted, where $T^{*}$ is the number of observations excluding the offending data points ^1^. The standard error $se\left( \hat{\beta}_{s,A1} \right)$ can be estimated by fitting unconstrained linear model(s) ^2^. The APC difference between two consecutive segments, using linearization approximation, is: ${APC}_{s+1,A}-{APC}_{s,A}\approx100\hat{\delta}_{As}$, where $s\geq1$.

An approximate lower and upper bounds of the 95% confidence interval for the ${AAPC}_{A}$ are:

${AAPC}_{A\_Lper}=100\left\{ exp\left[ log\left( \left( {{AAPC}_{A}}/{100} \right)+1 \right)-z_{0.975}\sqrt{\sum\tilde{\gamma}_{s}^{'}\hat{V}\left( \hat{\beta}_{1s} \right)\tilde{\gamma}_{s}} \right]-1 \right\}$ and

${AAPC}_{A\_Uper}=100\left\{ exp\left[ log\left( \left( {{AAPC}_{A}}/{100} \right)+1 \right)+z_{0.975}\sqrt{\sum\tilde{\gamma}_{s}^{'}\hat{V}\left( \hat{\beta}_{1s} \right)\tilde{\gamma}_{s}} \right]-1 \right\}$,

respectively, where $\tilde{\gamma}_{s}={\gamma_{s}}/{\sum\gamma_{s}}$, i.e., the normalized weight vector, $\hat{V}\left( \hat{\beta}_{1s} \right)$ is the estimated full variance-covariance matrix of $\hat{\beta}_{1s}.$

1. **Grid search default rules for model selection with given number of joinpoint**

The default rules include:

- - There is a minimum of three time points from the first and last joinpoint at either end of the span of the data along the x-axis of time (including the joinpoint).
  - There is a minimum of four time points between any two successive joinpoints (including the joinpoints).
  - It only allows joinpoints to be at the observed time points.

The first two rules are implemented to allow for estimation of the standard errors of the estimated regression coefficients, while the third rule is for computational simplicity. It’s worth noting that the National Cancer Institute’s (NCI) Joinpoint software allows users to add grid points between the observed time points. We also follow the default maximum number of joinpoints recommended by the NCI’s Joinpoint software at the grid search step ^3,4^.

These rules were determined based on whether the joinpoints have meaningful interpretation. For example, we want to fit joinpoint model with the 1991-2016 National Health Interview Survey (NHIS) data. Since there are 26 data points (years), based on the default rule, the maximum number of joinpoints being allowed is 4. Thus, at the grid search step, we will do the following:

- For jp1 models, test all single jointpoint locations 1993, 1994, …, 2014.
- For jp2 models, test all possible joinpoint location pairs between 1993 to 2014 that are at least 3 years apart, e.g., (1993, 1996), (1994, 1997), etc.
- For jp3 models, test all possible combination of three joinpoint locations between 1993 to 2014 that are at least 3 years apart between two adjacent joinpoints, e.g., (1993, 1996, 1999), (1993, 1996, 2000), etc.
- For jp4 models, test all possible combination of 4 joinpoint locations triples between 1993 to 2014 that are at least 3 years apart between two adjacent joinpoints, e.g., (1993, 1996, 2000, 2003), (1993, 1996, 2000, 2004), etc.

1. **Review of parameter and variance estimation for generalized linear models**

Following the similar development in SUDAAN 11 Language Manual in Section 4.8 ^5^, for the same design described in Section 2 of the main text, let E($y_{ijl})=\mu_{ijl}$, where $\mu_{ijl}$ is the population value, $g$ is the link function (e.g., identity, log or logit for linear, Poisson or logistic regression, respectively) with the relationship:

$g\left( \mu_{ijl} \right)=X_{ijl}^{'}\boldsymbol{\beta}$ and $\mu_{ijl}=g^{-1}\left( X_{ijl}^{'}\boldsymbol{\beta} \right){=g}^{-1}\left( L_{ijl} \right)$, (1)

where $L_{ijl}=X_{ijl}^{'}\boldsymbol{\beta}$ and $g^{-1}$ is the inverse of the link function, $\boldsymbol{\beta=}\left( \beta_{0}\boldsymbol{,}\beta_{1}\boldsymbol{,\ldots,}\beta_{p} \right)$ regression parameters with intercept $\beta_{0}$, $X_{ijl}$is a $\left( p+1 \right)$-dimensional vector consisting of the intercept and a $p\times1$ covariate matrix of independent variables, where the first value of the vector is 1, representing the intercept. The variance of $y_{ijl}$ is allowed to be function of $\mu_{ijl}$ denoted as:

$Var\left( y_{ijl} \right)=V\left( \mu_{ijl} \right)$.

We only consider the case where $y_{ijl}$ is a scalar and the working correlation matrix is identity. In this case, to estimate the parameter $\boldsymbol{\beta,}$ we can use the following estimating equation:

$\sum_{i=1}^{I} \sum_{j=1}^{J_{i}} \sum_{l=1}^{n_{ij}} \left\{ {\frac{\partial\mu_{ijl}}{\partial\boldsymbol{\beta}}w}_{ijl}\left[ V\left( \mu_{ijl} \right) \right]^{-1}\left( y_{ijl}-\mu_{ijl} \right) \right\}=0$. (2)

Using the relationship of equation (1),

$$\frac{\partial\mu_{ijl}}{\partial\boldsymbol{\beta}}=\frac{\partial g^{-1}\left( L_{ijl} \right)}{\partial L_{ijl}}\frac{\partial L_{ijl}}{\partial\boldsymbol{\beta}}=\frac{\partial\mu_{ijl}}{\partial L_{ijl}}X_{ijl},$$

The left-hand side of equation (2) is referred to as a score function and only depends on $\boldsymbol{\beta}$. The corresponding score function for the parameter $\beta_{k}$ in equation (2) is:

$$S\left( \beta_{k} \right)=\sum_{i=1}^{I} \sum_{j=1}^{J_{i}} \sum_{l=1}^{n_{ij}} \left\{ \frac{\partial\mu_{ijl}}{\partial L_{ijl}}X_{ijl}(k){w_{ijl}\left[ V\left( \mu_{ijl} \right) \right]}^{-1/2}\left( y_{ijl}-\mu_{ijl} \right) \right\}$$

To solve equation (2), a modified Newton Raphson method can be used. Let $\beta_{k}^{(m)}$ be the solution at the $m^{th}$ iteration, then the solution at the ${(m+1)}^{th}$ iteration is defined as:

$\beta_{k}^{(m+1)}=\beta_{k}^{(m)}-S\left( \beta_{k}^{(m)} \right)\left[ \frac{\partial S\left( \beta_{k}^{(m)} \right)}{\partial\beta_{k}} \right]^{-1}$,

Or in matrix notation,

$$\boldsymbol{\beta}^{(m+1)}=\boldsymbol{\beta}^{(m)}-J_{0}^{-1}S\left( \boldsymbol{\beta}^{\left( m \right)} \right),$$

where $\boldsymbol{J}_{0}$ is the R x R Jacobian matric whose ${(i,j)}^{th}$ element is defined as $S\left( \beta_{i}^{(m)} \right)/\partial\beta_{j}$ *,* and $S\left( \boldsymbol{\beta}^{\left( m \right)} \right)$ is a (p+1)-dimensional vector with elements $S\left( \beta_{k}^{(m)} \right),$*for* $k=0,1,\ldots,p.$ The iterations are carried out until convergence is achieved.

Let $\hat{\boldsymbol{\beta}}$denote the estimate of $\boldsymbol{\beta.}$The estimated robust variance-covariance of $\hat{\boldsymbol{\beta}}$ using the Binder (1983) method is given by:

$\hat{V}_{Binder}\left( \hat{\boldsymbol{\beta}} \right)=\left( J_{0} \right)^{-1}\hat{V}ar\left[ S\left( \hat{\boldsymbol{\beta}} \right) \right]\left( J_{0}^{'} \right)^{-1}$*,* (3)

where $\hat{V}ar\left[ S\left( \hat{\boldsymbol{\beta}} \right) \right]$ is the design-based variance of the score function.

Given the estimate $\hat{\boldsymbol{\beta}}$, the score functions are linear functions of the observations and may be written as:

$S\left( \beta\right)=\sum_{i=1}^{I} \sum_{j=1}^{J_{i}} \sum_{l=1}^{n_{ij}} A_{ijl}\left( y_{ijl}-\mu_{ijl} \right)$, where

$A_{ijl}={\frac{\partial\mu_{ijl}}{\partial L_{ijl}}X_{ijl}(k)w}_{ijl}\left[ V\left( \mu_{ijl} \right) \right]^{-1}$.

The estimated variance of the score function can be computed by using the Taylor linearization method for estimating the variance of a total for the given sample design.

The estimated model-based variance-covariance ^6^ is computed as:

$\hat{V}_{model}\left( \hat{\boldsymbol{\beta}} \right)={\phi I}_{0}^{-1}$, (4)

where $I_{0}=\sum_{i=1}^{I} \sum_{j=1}^{J_{i}} \sum_{l=1}^{n_{ij}} \left\{ \left( \frac{\partial\mu_{ijl}}{\partial\boldsymbol{\beta}} \right)w_{ijl}\left[ V\left( \mu_{ijl} \right) \right]^{-1}\left( \frac{\partial\mu_{ijl}}{\partial\boldsymbol{\beta}} \right)^{'} \right\}$ and the dispersion parameter $\phi$ depends on the model as well as the sample design. For more details, we refer to SUDAAN 11 Language manual ^5^.

1. **Lumley & Scott’s modified AIC**

Given the design described in Section 2 of the main text, the modification of the classical Akaike information criterion (dAIC) for linear model selection proposed by Lumley & Scott ^7^ to account for the complex survey design can be written as:

$dAIC=-2n\hat{l}\left( \hat{\boldsymbol{\beta}} \right)+2p\hat{\bar{\delta}}$, (5)

where the estimated population likelihood $\hat{l}\left( \hat{\boldsymbol{\beta}} \right)=\frac{1}{N}\sum_{i=1}^{I} \sum_{j=1}^{J_{i}} \sum_{l=1}^{n_{ij}} w_{ijl}l_{ijl}(\hat{\boldsymbol{\beta}})=\frac{1}{N}\sum_{i=1}^{I} \sum_{j=1}^{J_{i}} \sum_{l=1}^{n_{ij}} w_{ijl}log\left[ f_{\hat{\boldsymbol{\beta}}}(y_{ijl}|x_{ijl}) \right]$, $f_{\boldsymbol{\beta}}\left( y_{ijl} | x_{ijl} \right)$ is the likelihood of observation *l* in cluster *j* and stratum *i*, $N$ is the target population total or an estimate of it such as $\sum_{i=1}^{I} \sum_{j=1}^{J_{i}} \sum_{l=1}^{n_{ij}} w_{ijl}.$ $\hat{\bar{\delta}}=trace\left\{ {\hat{J}(\hat{\boldsymbol{\beta}}\text{)}}^{-1}\hat{V}(\hat{\boldsymbol{\beta}}) \right\},$ where $\hat{J}(\hat{\boldsymbol{\beta}}\text{)=}I_{0}^{-1}$ is the model-based (or simple random sample) variance-covariance matrix associated with $\hat{\boldsymbol{\beta}},$ $\hat{V}(\hat{\boldsymbol{\beta}}\text{)}$ is the full variance-covariance matrix under the complex design, defined by formula (3). The first row and column which correspond to $\hat{\beta}_{0}$ were removed from the two matrices. Note that dispersion parameter $\phi$ is not needed in the computation of $\hat{J}\left( \hat{\boldsymbol{\beta}} \right).$Here $\hat{\bar{\delta}}$ is the estimated average design effect and is used to inflate the penalty term $2p$ used for simple random samples.

1. **Estimation of** $\hat{\bar{\boldsymbol{\delta}}}$

To estimate $\hat{\bar{\delta}}$, for the constrained model, we can fit the constrained joinpoint model (2) and (3) from the main text, conditional on the $k$ estimated joinpoints, and then obtain the estimated variance $\hat{J}(\hat{\theta}\text{)}$ and $\hat{V}(\hat{\theta}\text{)}$ using R *svyglm* with options *naïve.cov* and *vcov*. Alternatively, SUDAAN procedures (*PROC REGRESS* and *PROC RLOGIST*) can also be used to compute the model-based variance $\hat{J}(\hat{\theta}\text{)}$ (with the *MODELVAR option*) and the robust variance $\hat{V}(\hat{\theta}\text{)}\text{ }$(with the *COVAR* option). However, it’s important to note that the model-based variance $\hat{J}(\hat{\theta}\text{)}$ from SUDAAN includes a dispersion parameter $\phi$which is not necessary for this context (as clarified by Thomas Lumley in Lumley & Scott 2015 and personal communication on 10/09/2020). Therefore, we use the *svyglm* in R to obtain the correct $\hat{J}(\hat{\theta}\text{)}$ (from the *naïve.cov* option). Following ^7^, we remove the 1^st^ row and the 1^st^ column (corresponding to the intercept) from both $\hat{J}(\hat{\theta}\text{)}$ and $\hat{V}(\hat{\theta}\text{)}\text{.}$ This results in modified matrices $\hat{\boldsymbol{J}}(\hat{\theta}\text{)}$ and $\hat{\boldsymbol{V}}(\hat{\theta}\text{)}$ that are $\boldsymbol{(}k+1)\times(k+1)$ rather than $\boldsymbol{(}k+2)\times(k+2)$. $\hat{\bar{\delta}}$ can then be estimated using the trace of ${\hat{\boldsymbol{J}}(\hat{\theta}\text{)}}^{-1}\hat{\boldsymbol{V}}(\hat{\theta})$.

1. **Data Generation and sampling for the Simulation study**

Our objective was to generate 20 years of data (t=1997-2016) for a continuous outcome and for a binary outcome separately, mimicking the body mass index variable and the obesity variable in NHIS respectively. For simplicity, we didn’t consider stratification in this study, so the number of strata was 1.

The finite target population (FTP) size was set to one million individuals. The FTP of individuals was partitioned into 1000 clusters with each cluster consisting of 1000 individuals. Eighty clusters were sampled from each year, with the sampled clusters being the same across the first 10 years, and then another set of sampled clusters being used across the second 10 years. For each year, individuals were sampled within each sampled clusters, with the same or varying sample rate. The details on the data generation and sampling are provided below.

*Step 1*: Generate yearly population means and proportions

*For continuous outcome*: FTP yearly means were generated using:

$E\left( log(u^{(t)}) \right)=\beta_{0}+\beta_{1}t+\delta_{1}\left( t-\tau_{1} \right)I_{\tau_{1}}(t$),

where $I_{\tau_{1}}(t)=1$ if $t>\tau_{1}$; *t* =1997-2016, $\tau_{1}=2003$, $\beta_{0}=-6.57705; \beta_{1}=0.00492,$ $\delta_{1}=(0, -0.0041, -0.041)$.

The corresponding true values of the *APC* pair for the two segments before and after the joinpoint were:

$\left( 0.492\%, 0.492\% \right),$when $\delta_{1}=0;$

$\left( 0.492\%, 0.082\% \right),$ when $\delta_{1}=-0.0041;$ and

$\left( 0.492\%, -3.608\% \right)$ when $\delta_{1}=-0.041.$

The corresponding approximate *APC* differences between the two segments before and after the joinpoint for the three scenarios are: ($0, -0.41\%, -4.1\%$).

*For binary outcome*: FTP yearly proportions $P^{(t)}$were generated using

$logit(P^{(t)}{)=\beta}_{0}+\beta_{1}t+\delta_{1}\left( t-\tau_{1} \right)I_{\tau_{1}}(t$);

where *t* =1997-2016, $\tau_{1}=2003$, $\beta_{0}=-103.8; \beta_{1}=0.00512,$ $\delta_{1}=(0, -0.00485, -0.0485)$. We also tested a larger $\delta_{1}$ value -0.285.

The corresponding *OAPC* for the two segments before and after the joinpoint were: $\left( 0.512\%, 0.512\% \right),$when $\delta_{1}=0;$

$\left( 0.512\%, 0.027\% \right),$when $\delta_{1}=-0.00485;$

$\left( 0.512\%, -4.288\% \right)$, when $\delta_{1}=-0.0485;$ and

$\left( 0.512\%, -27.99\% \right)$ when $\delta_{1}=-0.285.$

The corresponding approximate *OAPC* differences between the two segments before and after the joinpoint are: ($0, -0.485\%, -4.85\%, -28.5\%$).

*Step 2*: Generate clusters and individual outcomes within each cluster across years

Our goal was to generate outcomes for individuals in each FTP cluster using normal distribution for continuous outcome and Beta-Bernoulli for binary data where three sets of intra-cluster correlation coefficient (*ICC*) values $ICC=0, 0.01, 0.075$ were used based on practical considerations. We also tested a larger ICC value 0.3, though in our clustering design setting large ICC values like 0.3 corresponds to non-practical large design effect.

To generate *continuous* outcome, we first generated a cluster random effect $v_{i}\sim Normal (0, \varphi^{2})$, $i=1,\ldots,1000$, with $\varphi=(0, 0.01848, 0.052358, 0.12037)$ which corresponds to the three ICC values (0, 0.01, 0.075, 0.3). For each $\varphi$ value, for each year within each cluster, we then generated 1000 observations with an outcome $logy_{ijt}=log\mu_{t}+v_{i}+u_{ijt}$, where $u_{ijt}$ is generated using $u_{ijt}\sim Normal (0, \sigma^{2})$, and $\sigma=$0.183875.

To generate *binary* outcome, we first generated yearly population proportions $P_{j}^{(t)}$ for each cluster using $P_{j}^{(t)}\sim Beta (a, b)$, where $a=P^{(t)}(1-\rho)/ \rho$ , $b=\frac{\left( 1-P^{(t)} \right)\left( 1-\rho\right)}{\rho},$ $\rho$ is the desired ICC,$\rho=(0.000001, 0.01, 0.075)$ which corresponds to the three ICC values (0, 0.01, 0.075). We then generated 1000 observations with a binary outcome$y_{jl}^{(t)}\sim Bernoulli\left( P_{j}^{\left( t \right)}, N_{j}^{\left( t \right)} \right),$where $N_{j}^{\left( t \right)}=1000.$

*Step 3:* Sampling clusters

To mimic the NHIS sample design, the sampling of the FTP clusters overlapped across years. We first randomly drew 80 clusters from the 1000 clusters for year 1997, then used the same set of sampled clusters for years 1998 to 2006. We redraw 80 clusters for year 2007 and used the same set of sampled clusters for years 2008 to 2016.

*Step 4:* Sampling individuals within sampled clusters

When sampling within sampled clusters, we wanted to evaluate the effect of sample size (small, medium and large) on the selection results. Therefore, within each year an independent random sample of individuals with their outcomes were selected with pre-defined sampling rates of $r_{j}=5\%,$20% or 50%. The total sample size from each year was 4000 (= $0.05\times1000\times80$), 16,000 (= $0.2\times1000\times80$) and 40,000 (= $0.5\times1000\times80$) for the three scenarios. As stated, the within cluster sampling are independent from cluster to cluster and year to year. Sample weights for each individual sample $l$in cluster $j$ in year $t$ were calculated as the inverse of the sampling probability, i.e., $w_{jl}^{(t)}=\frac{1}{r_{l}}.$ We used equal probability sampling in this experiment for simplicity. With APC difference of -4.10%, we also tested sampling with a varying sampling rate across the clusters (random draw from a uniform distribution from interval (5%, 15%), so the mean sampling rate was 10% for each ICC value case.

*Step 5:* Replication

For each data scenario, depending on the outcome (continuous or binary), ICC value (0, 0.01, 0.075, 0.3), APC difference ($0, -0.41\%, -4.1\%$) for continuous outcome or OAPC difference ($0, -0.485\%, -4.85\%, -28.5\%$), and sampling rate (5%, 20%, 50% and varying sampling rate with average 10%) (note that varying sampling rate was only applied when APC difference=-4.1% or OAPC difference=$-4.85\%$or $-28.5\%$), step 3 and step 4 were repeated 100 times to form 100 samples, each sample contains 20 years of data.

**References**

1. Kim H-J, Yu B, Feuer EJ. Inference in segmented line regression: A simulation study. *Journal of Statistical Computation and Simulation.* 2008;78(11):1087-1103.

2. National Cancer Institute. Annual Percent Change (APC) and Confidence Interval. <https://surveillance.cancer.gov/help/joinpoint/setting-parameters/method-and-parameters-tab/apc-aapc-tau-confidence-intervals/estimate-average-percent-change-apc-and-confidence-interval>. Accessed Dec 2, 2024.

3. National Cancer Institute. Joinpoint Trend Analysis Software: Number of Joinpoints. <https://surveillance.cancer.gov/help/joinpoint/setting-parameters/method-and-parameters-tab/number-of-joinpoints>. Accessed Nov 8, 2024.

4. National Cancer Institute. Joinpoint Trens Analysis Software: New default settings for Grid Search. <https://surveillance.cancer.gov/help/joinpoint/tech-help/frequently-asked-questions/new-default-settings-for-grid-search>. Accessed Nov 8, 2024.

5. Research Triangle Institute. SUDAAN Language Manual, Volumns 1 and 2, Release 11. *Research Triangle Park, NC: Research Triangle Institute.* 2012.

6. Zeger SL, Liang KY. Longitudinal data analysis for discrete and continuous outcomes. *Biometrics.* 1986;42(1):121-130.

7. Lumley T, Scott A. AIC and BIC for modeling with complex survey data. *Journal of Survey Statistics and Methodology.* 2015.

Table S1: Kish’s design effect associated with different sampling designs in the simulation settings

| cluster size in FTP | sampling rate | Average sample cluster size | ICC | DEFF |
| --- | --- | --- | --- | --- |
| 1000 | 5% | 50 | 0 | 1 |
| 1000 | 20% | 200 | 0 | 1 |
| 1000 | 50% | 500 | 0 | 1 |
| 1000 | Unif (5%,15%)(average 10%) | 100 | 0 | 1 |
| 1000 | 5% | 50 | 0.01 | 1.49 |
| 1000 | 20% | 200 | 0.01 | 2.99 |
| 1000 | 50% | 500 | 0.01 | 5.99 |
| 1000 | Unif (5%,15%)(average 10%) | 100 | 0.01 | 1.99 |
| 1000 | 5% | 50 | 0.075 | 4.675 |
| 1000 | 20% | 200 | 0.075 | 15.925 |
| 1000 | 50% | 500 | 0.075 | 38.425 |
| 1000 | Unif (5%,15%)(average 10%) | 100 | 0.075 | 8.425 |
| 1000 | 5% | 50 | 0.3 | 15.7 |
| 1000 | 20% | 200 | 0.3 | 60.7 |
| 1000 | 50% | 500 | 0.3 | 150.7 |
| 1000 | Unif (5%,15%)(average 10%) | 100 | 0.3 | 30.7 |

*Note*: Design effects bigger than 15 are highlighted in grey. FTP: Finite target population.

Table S2: Percent (%) of samples correctly identified true number of joinpoint(s) for a continuous outcome: log-normal models.

| sampling rate | APC difference | ICC=0.3 | | | |
| --- | --- | --- | --- | --- | --- |
|  |  | aggregate-level model | | individual-level model | |
|  |  | without COV | with COV | constrained | unconstrained |
| 5% (n=4000) | 0.00% | 57 | 95 | **99** | 0 |
|  | -0.41% | 58 | **100** | 18 | 27 |
|  | -4.10% | 49 | **100** | 98 | 10 |
| 20% (n=16,000) | 0.00% | 25 | 95 | **99** | 0 |
|  | -0.41% | 43 | **95** | 20 | 31 |
|  | -4.10% | 29 | 95 | **98** | 9 |
| 50% (n=40,000) | 0.00% | 19 | 90 | **99** | 0 |
|  | -0.41% | 35 | **91** | 21 | 30 |
|  | -4.10% | 19 | 91 | **98** | 6 |
| uniform 5%~15% (average n=8,000) | 0.00% | 40 | 93 | **99** | 0 |
|  | -0.41% | 60 | **99** | 23 | 27 |
|  | -4.10% | 44 | **99** | 98 | 9 |

*Note*: The largest percentage in each row is bolded. When the true APC difference is none zero but very small (-0.41%), most runs using the constrained unit-level models chose zero-joinpoint model, some of the unconstrained unit-level models chose joinpoint model with either two or three joinpoints.

Table S3: Percent (%) of replicates correctly identify true number of joinpoint(s) for a binary outcome: log-normal for aggregate-level proportion and individual-level logistic models.

| sampling rate | OAPC difference | ICC=0.3 | | | |
| --- | --- | --- | --- | --- | --- |
|  |  | aggregate-level model | | Individual-level model | |
|  |  | without COV | with COV | constrained | unconstrained |
| 5% (n=4,000) | 0 | **86** | 85 | 82 | 82 |
|  | -0.485% | 4 | 1 | 9 | **12** |
|  | -4.85% | 35 | 37 | **49** | 46 |
|  | -24.25% | 94 | **100** | 80 | 81 |
| 20% (n=16,000) | 0 | 89 | **91** | 81 | 83 |
|  | -0.485% | 4 | 3 | 14 | **15** |
|  | -4.85% | 36 | 33 | **49** | 45 |
|  | -24.25% | 95 | **100** | 78 | 79 |
| 50% (n=40,000) | 0 | 89 | **92** | 80 | 80 |
|  | -0.485% | 3 | 1 | **15** | 13 |
|  | -4.85% | 33 | 32 | 47 | **48** |
|  | -24.25% | 94 | **100** | 80 | 82 |
| uniform  5%~15%  (average n=8,000) | 0 | **91** | 87 | 82 | 80 |
|  | -0.485% | 6 | 2 | 12 | **13** |
|  | -4.85% | 32 | 28 | **52** | 48 |
|  | -24.25% | 93 | **94** | 77 | 78 |

*Note*: The largest percentage in each row is bolded. When OAPC difference is -0.485%, majority of the runs picked the model with zero joinpoint. ICC: intra-cluster correlation coefficient.
